# Three Draft Single-Cell Genome Sequences of Novel SAR324 Strains Isolated from the Abyssopelagic Southern Ocean

**DOI:** 10.1128/MRA.00759-21

**Published:** 2021-09-30

**Authors:** Diego J. Castillo, Marc W. Van Goethem, Thulani P. Makhalanyane

**Affiliations:** a Centre for Microbial Ecology and Genomics (CMEG), Department of Biochemistry, Genetics and Microbiology, University of Pretoria, Pretoria, South Africa; Montana State University

## Abstract

SAR324 is a ubiquitous and phylogenetically distinct clade of Deltaproteobacteria in marine environments. Here, we present three single-cell amplified genome sequences from the SAR324 lineage, obtained from the abyssopelagic zone of the Indian sector of the Southern Ocean.

## ANNOUNCEMENT

Members of SAR324 mediate important biogeochemical processes in the oceans (1–6). However, the lack of sequence representatives of this clade limits efforts to gain a mechanistic understanding of their precise functional roles (2, 3). Members of SAR324 have been found throughout the water column (4, 5) and are metabolically versatile (2, 6). Current physiological insights regarding this clade are derived primarily from environmental samples, based on 16S rRNA gene surveys (5), metagenomics and metatranscriptomics (2, 3, 7–9), and single-cell genomics (6, 10). Here, we present three SAR324 genome sequences, obtained from the abyssopelagic zone of the Southern Ocean using single-cell genomics.

A water sample was collected at a depth of 4,154 m in the Indian sector of the Southern Ocean (47.994°S, 37.034°E) and preserved as detailed previously (11). Fluorescence-activated cell sorting and multiple displacement amplification were performed at Bigelow Laboratory for Ocean Sciences (ME, USA), as previously described (11). Single-cell amplified genomes (SAGs; *n* = 41) were selected for library preparation using the Nextera XT DNA kit, according to the manufacturer’s instructions. The libraries were sequenced at Admera Health, LLC (NJ, USA) using an Illumina HiSeq X sequencer (150-bp paired-end reads). Bioinformatics analysis was conducted using KBase (12). The raw reads were processed using Trimmomatic v0.36 (13) and assembled using SPAdes v3.13.0 (14), with “single-cell” entered as the DNA source. The assemblies were evaluated using QUAST (15), while the SAG completeness and contamination were estimated using CheckM v1.018 (16). The assembly quality was determined using minimum information about a single amplified genome (MISAG) standards (17). The genomic coverage was calculated using BBTools (18). The genome statistics are provided in Table 1. Genome Taxonomy Database Toolkit (GTDB-Tk) v1.1.0 release 89 (19) was used to assign taxonomy, and protein-encoding regions were identified using Prokka v1.14.5 (20). Average nucleotide identities (ANI) of reciprocal hits were calculated between our three SAR324 SAGs and against two SAR324 draft genome sequences: SAR324 bacterium lautmerah1 (3) and SAR324 Arctic96AD-7 (genome 046) (9) (http://enve-omics.ce.gatech.edu/ani/). Finally, we compared the 16S rRNA gene of each SAG to all 16S rRNA gene sequences available in the NCBI nonredundant (nr) database. Default parameters were used for all software unless otherwise noted.

**TABLE 1:** Genome statistics and comparisons of three potentially novel SAR324 SAGs

Characteristic	Data for strain:		
	SAR324_I22	SAR324_K2	SAR324_N8
No. of raw paired-end reads	11,549,492	8,050,736	10,928,754
No. of quality-filtered paired-end reads	10,024,104	6,997,348	9,413,936
Assembly size (bp)	2,453,850	2,467,220	2,070,041
Coverage (×)	601	422	549
G+C content (%)	44.72	41.42	42.43
Estimated genome completeness (%)	56.15	50.71	50.14
Predicted genome size (bp)	4,370,169	4,865,352	4,128,522
Estimated contamination (%)	3.2	3.36	3.03
Genome quality	Medium	Medium	Medium
No. of contigs	707	505	646
Largest contig (bp)	87,181	87,899	52,735
*N*_50_ (bp)	13,771	23,927	11,591
No. of protein-coding genes	2,346	2,337	1,952
No. of tRNA genes	31	32	19
No. of rRNA genes	8	3	3
ENA raw read accession no.	ERR6548281	ERR6548282	ERR6548383
ENA assembly accession no.	CAJYYN010000000.1	CAJYYM010000000.1	CAJYYL010000000.1
ANI with bacterium lautmerah1 (%)	74.02	80.96	73.11
ANI with Arctic96AD-7 (%)	79.37	97.01	97.54
16S rRNA gene highest similarity isolate name	*Pseudomonadaceae* SI-3	Uncultured delta proteobacterium	Uncultured bacterium clone
GenBank accession no.	CP026511.1	GU474888.1	HQ674466.1
Similarity (%)	100	99.87	99.79

Based on current standards (17), our three SAR324 genome sequences were classified as medium-quality draft genome sequences and were taxonomically assigned as deltaproteobacterial group SAR324 (strain Arctic96AD-7). SAR324_K2 and SAR324_N8 had the highest genome similarity, with 96.75% ANI. Both genomes were distinct from SAR324_I22 (ca. 81.9% ANI), suggesting that they may have different identities at the species or genus level, within the same family. ANI percentages between our three SAR324 SAGs and two SAR324 reference genomes (3, 9) and comparisons of the 16S rRNA gene sequences available in the NCBI (Table 1) revealed substantial differences within this versatile group. Metabolic pathway reconstructions showed evidence for carbon fixation (phosphoribulokinase; *prk*) in SAR324_N8. Indicator genes for sulfur oxidation (*soxAB*) were found in both SAR324_I22 and SAR324_K2 (3 copies of *soxB*) but not in SAR324_N8.

### Data availability.

The genome assemblies for the three SAGs have been deposited at the ENA under accession number PRJEB47084, and the accession numbers are given in Table 1.
